# Supplementary material for: Antioxidant Responses Induced by PFAS Exposure in Freshwater Fish in the Veneto Region
Source: Antioxidants (Basel). 2022 Jun 3;11(6):1115. doi: 10.3390/antiox11061115 (PMC9219832; doi:10.3390/antiox11061115)
Supplement: Supplementary file 1 [file antioxidants-11-01115-s001.zip › antioxidants-1743281-supplementary.pdf]

Table S1. Primer pairs used for qRT-PCR. Amplicon sizes and annealing temperatures (Ta) are also indicated.

PRIMER	Sequences 5′→3′	Amplicon (bp)	Ta (°C)
3_FW_SOD2_GOBY_RT	5'- ACAAGATGTCTGCCGCCACA-3'	162	60
3_REV_SOD2_GOBY_RT	5'- GCTCCCACACGTCAATGCCC-3'		60
go_GPX4b_b_fw	5′- TTGGAAGTGGATGAAGGCTC-3′	134	60
go_GPX4b_b_rv	5′-CACTTGGATCATCTGTGGGG-3′		60
sq_SOD2_b_fw	5′-CACTACAGGTCTCGTCCCAC-3′	116	60
sq_SOD2_b_rv	5′-CCCAGCTCACAACATTCCAG-3′		60
1_FW_GPX4_CAV_RT	5'- AGCGGACATAAAGGAGTTTGCT-3'	199	60
1_RV_GPX4_CAV_RT	5'- CCTCTTCACGACCCGACCTT-3'		60
sq_GAPDH_b_fw	5′-ATCACAGCCACACAGAAGAC-3′	126	60
sq_GAPDH_b_rv	5′-AGGAATGACTTTGCCCACAG-3′		60
